# Examination of Human Health Impacts Due to Adverse Climate Events Through the Use of Vulnerability Mapping: A Scoping Review

**DOI:** 10.3390/ijerph16173091

**Published:** 2019-08-26

**Authors:** Michael T. Schmeltz, Peter J. Marcotullio

**Affiliations:** 1 Department of Health Sciences, California State University, East Bay, Hayward, CA 94542, USA; 2 Department of Geography, Hunter College, City University of New York (CUNY), New York, NY 10065, USA; 3 City University of New York Institute for Sustainable Cities, New York, NY 10065, USA

**Keywords:** climate change, public health, vulnerability mapping, health outcomes, geospatial analysis, extreme heat, flooding, vector-borne disease, wildfire

## Abstract

Government officials, health professionals, and other decision makers are tasked with characterizing vulnerability and understanding how populations experience risks associated with exposure to climate-related hazards. Spatial analyses of vulnerable locations have given rise to climate change vulnerability mapping. While not a new concept, the spatial analyses of specific health outcomes remain limited. This review explores different methodologies and data that are used to assess vulnerability and map population health impacts to climate hazards. The review retrieved scholarly articles and governmental reports concerning vulnerability mapping of human health to the impacts of climate change in the United States, published in the last decade. After review, 37 studies were selected for inclusion. Climate-related exposures were distributed across four main categories, including: high ambient temperatures; flood hazards; vector-borne diseases; and wildfires. A number of different methodologies and measures were used to assess health vulnerability to climate-related hazards, including heat vulnerability indices and regression analyses. Vulnerability maps should exemplify how variables measuring the sensitivity and adaptive capacity of different populations help to determine the potential for climate-related hazards to have an effect on human health. Recommendations address methodologies, data gaps, and communication to assist researchers and stakeholders in directing adaptations to their most efficient and effective use.

## 1. Introduction

Key to the concept of vulnerability is developing an understanding of how populations experience health related impacts due to climate change. Social geography, economics, ecology, public health, and the physical sciences all have contributed to the definition of vulnerability and have different interpretations of the term. Some definitions of vulnerability only examine causal mechanisms to identify vulnerable populations (e.g., exposure to extreme heat) while others incorporate ideas of coping, mitigation, and recovery. Blaikie et al. [1] defines vulnerability as, “the characteristics of a person or group and their situation that influence their capacity to anticipate, cope with, resist and recover from the impact of a natural hazard”. The Intergovernmental Panel on Climate Change (IPCC) defines climate vulnerability as the propensity or predisposition to be adversely affected by climate variability and change. The IPCC concludes that vulnerability to climate change will encompass certain factors, including exposure, susceptibility or sensitivity to an event, and the ability (or lack thereof) to improve the adaptive capacity of a response to identified risks to human health [2].

Other differences across definitions of vulnerability involve the categorization of exposures and risks used in those definitions. Some definitions look at a combination of exposure variables (environmental, social and geographical) while others only incorporate socioeconomic variables to measure risk. For example, some researchers identify populations vulnerable to coastal storms as those living below the poverty line, while others may define vulnerable populations as only those who live along rivers or coasts, where elevation is the key determinant to exposure [3]. These definitions of vulnerability also differ from that of risk, which, in terms of climate change, can be defined as the probability of harmful consequences resulting between hazards (climate related event) and vulnerable conditions (population sensitivity or susceptibility) [4]. Acknowledging that there is a range of definitions of vulnerability and risk is important as doing so helps us interpret research findings and their meaningful application to adaptation strategies [5,6,7]. For the purposes of this study, we focus on vulnerability and define it using the recent U.S. Climate and Health Assessment’s definition, as “the tendency or predisposition to be adversely affected by climate-related health effects, and encompasses three elements: exposure, sensitivity or susceptibility to harm, and the capacity to adapt to or to cope with change” [8]. In particular, this review focuses on health and how it is affected by climate change through examining the use of vulnerability mapping to assess exposure, sensitivity, and adaptive capacity and visualize how population health may be impacted by climate-related hazards. While projections of greenhouse gas (GHG) emissions are important to assess future climate and hence its potential impacts on populations, this review does not examine emission scenarios or projections of GHG concentrations.

Vulnerability assessments are important tools for identifying and increasing adaptive capacity and building resilience among vulnerable populations [9]. Government officials, public health practitioners, scientists, healthcare providers, first responders and other decision makers both use and create vulnerability assessments in an attempt to understanding how populations of concern experience risks associated with exposure to climate-related health hazards. A significant body of work related to the conduct of vulnerability assessments has focused on public health outcomes related to impacts from climate change that results in changes in morbidity, mortality, or risk perceptions [10,11,12]. Maps are often used to examine the vulnerability of a place using overlays of exposures and populations sensitive to health outcomes from climate-related hazards. The study refers to health outcomes, which broadly covers the physical, mental, and social well-being of an individual or population encompassing both biophysical and social determinants of health. It is used in lieu of more specific measures because many of the studies examined lacked clinical data on health impacts due to climate change.

Preston et al. [13] described spatial vulnerability assessments as possessing four key characteristics that can aid researchers and policy makers in engaging stakeholders. These four characteristics include visualization; local orientation; integration of social and biophysical determinants; and guidance for adaptation responses [13]. The integration of social and biophysical determinants (health outcomes) is a key characteristic that needs greater inclusion in the development of vulnerability maps that assess the human health impacts associated with climate change. The ability to produce vulnerability maps may be limited by data gaps and differing spatial scales and aggregation may be required to employ these methods [14]. For instance, health data can be analyzed through a variety of approaches, but personal health information is protected by law and accessing health data for secondary data analyses, like vulnerability mapping, can be thwarted by a complex web of ethical, social, and technological issues [15,16].

While vulnerability mapping is not a new concept [13,17]. In the United States, a significant research effort has resulted in vulnerability maps for heat morbidity and mortality [18,19,20,21]. At the same time, however, vulnerability health mapping in the United States for other climate hazards remains limited in the United States [22,23]. This data gap is important as analysts project climate change to result in increased the frequency and intensity of other extreme weather events including increases in the incidence of water-borne and vector-borne diseases. Moreover, the health outcomes of affected populations by varying climate hazards differs in important ways across income, racial and other groups and locations, demanding a full exploration of causes [24].

Unfortunately, there are a number of limitations associated with data availability and temporal inconsistencies that restrict the application and the usefulness of vulnerability maps [13,25]. There are also significant data gaps related to health outcomes at different resolutions or extents, which can inhibit our ability to identify, assess, and map vulnerable populations and health impacts [26]. These issues may be addressed, in part, by spatial downscaling or, in partnership with public health agencies, collecting relevant health outcome data. As technology improves and availability of geospatial data increases vulnerability mapping will have the ability to improve greatly. Federal and state governments have also increased access and usability of vulnerability mapping (e.g., U.S. Climate Resilience Toolkit and California’s Cal-Adapt). Although, to date, there has been limited direction or ‘best practices’ for mapping the vulnerability of human health to the impacts of climate change.

This review builds off a recent report from the U.S. Environmental Protection Agency on vulnerability mapping [27]. The goal of this study is to identify and describe the differing methodologies and data used to assess and map health-related vulnerability indicators associated with climate hazards in the United States. The aim is to provide background and recommendations to improve the creation and use of vulnerability mapping for health outcomes.

## 2. Methodology

A literature search was conducted to identify scholarly articles, governmental reports, and projects concerning climate change vulnerability mapping of human health in the United States, published between January 2008 and December 2018. The authors followed the guidelines of the PRISMA (Preferred Reporting Items for Systematic Reviews and Meta-Analyses) extension for scoping reviews to conduct the analysis [28]. Several online databases were queried, including Web of Science, PubMed, Science Direct, Scopus, and Google Scholar. The keywords “vulnerability mapping” and “climate change” were used as inclusion criteria for articles, reports, and projects in combination with the following terms: spatial analysis, GIS, health, illness, disease, disorder, disaster, mortality, morbidity, hospitalization, emergency, preparedness, adaptation, vulnerability assessment, exposure, sensitivity, and risk. The criteria for selection were articles that used spatial analyses to examine vulnerability and had an appropriate health risk measure (e.g., incidence of disease, hospitalization, or mortality) as an outcome. Also included were studies that spatially analyzed vulnerability through the identification of a vulnerable population based on geographic, socioeconomic, and demographic characteristics often described as a social vulnerability index. These indices include demographic and sociodemographic characteristics, such as age and race, and are considered proxies for health status [29,30]. Papers were restricted to geographic locations within the United States (including Puerto Rico). Search results were downloaded to an EndNote library where duplicates were removed. Titles and abstracts were screened for relevance and full texts were obtained for further assessment if papers met inclusion criteria. Articles without full text, in a language other than English, or without sufficient details about data, methods, maps, and analytical techniques were excluded. Studies were examined to determine variables that measured exposure, sensitivity, and adaptive capacity and how these elements were used to assess vulnerability to human health due to climate change.

## 3. Results

**Figure 1:**
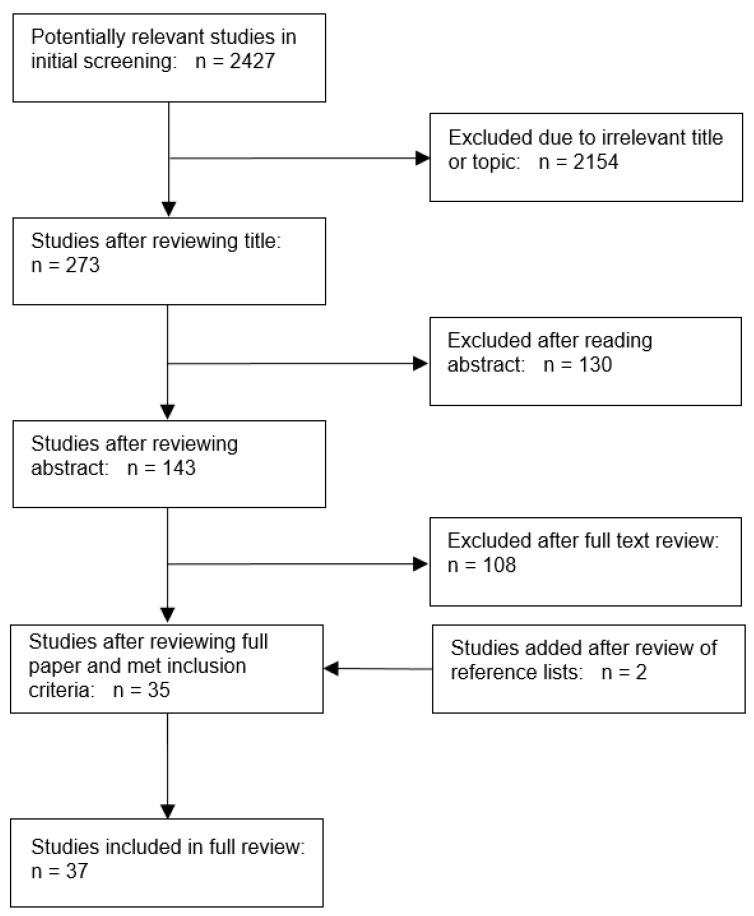
The literature selection process.

A total of 2427 papers were identified through the initial screening. From those, 37 papers, based in the United States, were included in the review (Figure 1). Table 1 summarizes the main characteristics of the 37 studies included in the final review. The climate-related exposures examined in the literature were described by four main categories: high ambient temperatures; flood hazards, including heavy precipitation events and sea-level rise; vector borne diseases and disease-causing micro-organisms, and wildfires. Each of these exposures focuses on how they assess health outcomes using vulnerability mapping.

### 3.1. High Ambient Temperature

The majority of the vulnerability mapping papers (26 of 37 (70.2%)) that examined human health outcomes focused on exposure to high ambient temperatures [15,18,19,20,21,31,32,33,35,36,39,40,41,43,44,45,46,47,48,52,54,55,57,59,60,61]. Many of these studies (18 of 26, (69%)) were in large urban areas, such as New York City, Philadelphia, Chicago, and Phoenix. The remaining papers examined multiple urban locations, entire states/territories, or the U.S. as a whole.

A variety of different data were accessed to measure the health impact of heat. Boumans et al. [32] and Uejio et al. [57] examined heat morbidity by using data from emergency calls and medical complaints made during extreme heat events. Kovach et al. [48], Prudent et al. [55], and the Wisconsin Department of Health Services [61] obtained hospitalization data on individuals diagnosed with a heat-related illness (ICD-9 codes 992.xx) to determine heat-related morbidity. The remaining papers used heat-mortality as an end-point for measuring health outcomes, determined by deaths associated with a defined heat event or deaths occurring above a pre-determined temperature threshold. There were a number of different temperature measures obtained from satellites (land-surface temperatures) and various weather stations (air temperatures), capturing minimum, mean, and maximum temperatures with some studies calculating a heat index or apparent temperature determined by the inclusion of humidity. No one temperature metric was adopted by all studies.

Variable selection for vulnerability mapping was mainly informed by a literature review that examined sociodemographic and environmental risk factors associated with heat morbidity and mortality. Variables came from case studies and regression analyses of hospitalizations and deaths occurring during and after extreme heat events. Many of the papers followed a methodology similar to that developed by Reid et al. [21] using principal component analysis (PCA) to calculate a heat vulnerability index. Factor analysis and PCA typically weight variables based on how much each contributes to determining variation in the dependent variable. Other studies weighted all variables equally. In all studies using factor analysis, variable scores were normalized so that a vulnerability index could be derived and mapped [31,35,52,59,60].

If the study did not deploy factor analysis and PCA, the analysts used a variety of other statistical methods for mapping vulnerability to high ambient temperatures. For example, Johnson et al. [19] and others [45,47,48,57] used different statistical regression methodologies (e.g., generalized linear mixed model and linear models) and directly mapped odds ratios, mortality rate ratios, or developed maps of high risk ‘hot spots’ overlaying spatial temperature data to identify vulnerable populations. The majority of the studies used sociodemographic variables to represent social determinants of health in developing indices to assess the health vulnerability associated with extreme heat without the use of direct health outcome variables (i.e., diagnosis of heat-related illness).

### 3.2. Sea-Level Rise, Flood Hazards and High Precipitation Events

Six papers (6 of 37 (16.2%)) examined health-related vulnerability mapping of flood hazards, including sea-level rise (SLR) and heavy precipitation events [34,50,51,53,55,58]. Four of the papers used a similar methodology for calculating a social vulnerability index (SoVI) to examine the human health impacts resulting from flooding [34,53,55,58]. While both Burton and Cutter [34] and Wang and Yarnel [58] used the Federal Emergency Management Agency’s (FEMA) 100-year flood zone to identify at-risk locations, Burton and Cutter [34] used FEMA’s HAzus model to map inundation risk while Wang et al. used the National Oceanic and Atmospheric Administration’s (NOAA) Sea, Lake and Overland Surges from Hurricanes (SLOSH) model for storm surge. Both papers looked at the intersection of vulnerable populations, as quantified by SoVI, with flood exposures (e.g., inundation maps). Martinich et al. [53] employed the SLR national coastal property model (NCPM) [63,64] to examine coastal inundation and similarly overlaid a mapped SoVI index to geographically represent vulnerable populations under three different projected emission scenarios (low, medium and high). Prudent et al. [55] also developed a vulnerability index, which included sociodemographic variables and variables from the build environment (watersheds). Unlike the other flood hazard studies, they used mortality data to determine a baseline measure for population health. The authors then linked the social-built environmental index score to the baseline mortality measures to determine where poor health, social marginalization, and the built environment increased vulnerability to flood hazards [55].

Liu et al. [50] used outcome measures from a household health vulnerability survey to examine emergency preparedness, financial ability to support recovery, and medical fragility. Similar to Wang and Yarnel [58], a SLOSH model was used to model the flood hazard and a fuzzy logic analysis to model vulnerability. Fuzzy logic can work with many factors at a variety of measurement scales and demonstrates advantages in dealing with multidimensional or complex conditions. Liu et al. [50] go into greater depth concerning their fuzzy logic [65,66,67] though for the purposes of this review their outcomes were similar to that of deriving a health vulnerability index, similar to the methodologies above.

Maantay et al. [51] used the FEMA 100-year flood zone to show the impact of flood hazards in New York City. A Human Hazard Vulnerability Index (HHVI) was calculated using sociodemographic variables to identify vulnerable populations. Another methodology (Cadastral-based Expert Dasymetric System—CEDS) which uses census data to estimate finer-scale resolution of at-risk populations was also developed to enhance the geographic extent and magnitude of vulnerability to flood hazards in New York City. By providing population density information, the human hazard index estimates the vulnerability of a population to flood hazards.

Only Prudent et al. [55] used health outcome variables to develop their index, though measures of adaptive capacity, such as access to medical care (e.g., distance to hospitals, number of physicians per 100,000 population) was included as a variable in most of the social vulnerability indices for assessing flood hazards. Liu et al. [50] also included specific questions on their household survey of medically fragile persons by measuring mobility, medical regimen, mental cognition, sensory impairment and assistance with activities of daily living.

### 3.3. Vector-Borne Disease and Infectious Micro-Organisms

Of the thirty-seven papers examined in the review, five (13.5%) were related to vulnerability mapping of health hazards associated with climate change and vector-borne diseases or infectious micro-organisms [37,42,49,56,62]. The papers examined vector-borne diseases such as West Nile Virus (WNV), plague, and infectious micro-organisms causing coccidioidomycosis, and liver disease. Vectors focused on mosquitos and rodents with infectious micro-organisms focusing on fungus and cyanobacteria. Analyses characterize the spread of these disease-carrying vectors due to a changing climate with associated changes in vector habitats, growth, and spore dissemination (specifically for coccidioidomycosis).

Holt et al. [42] used ecological niche modeling to develop a suitability index for the vector (e.g., mosquitos) and mapped the likelihood of disease transmission based on overlays of vector concentrations and human populations. This approach focused on the suitability of the habitat for the vector and the projected exposures due to the interaction of vectors and human populations that also relate to modelled predictions of changes in temperature and precipitation.

In their examination of WNV, Cleckner and Allen [37] used dasymetric mapping to transform data and better define patterns of vulnerability for human health. They quantified mosquito habitat and abundance and combined those factors with characteristics of human populations, particularly older adults and children, to identify ‘hotspots’. These hotspots were used to predict areas of higher WNV risk. Liu and Weng [49] also examined mosquito carriers of WNV based on vector habitat distribution. Data were collected from mosquito breeding pools that tested positive or negative for WNV and examined environmental factors that contributed to the penetration of mosquito populations in at-risk locations. Regression analyses were used to assess the importance of environmental variables in determining suitable habitats for mosquito testing. No diagnoses of WNV were used in the model and the focus was solely on suitability for vector habitat, which could determine risk of virus transmission to humans [49].

Zhang et al. [62] examined the association between cyanobacterial blooms and mortality due to non-alcoholic liver disease. Locations of cyanobacterial blooms (as measured by phycocyanin levels) were estimated via satellite. Non-alcoholic liver disease mortality was obtained from national mortality records and a flexible-shaped spatial scan statistic (FlexScan) was used to identify spatial clusters of liver disease. Exploratory spatial data analyses and Bayesian regression models were used to examine the association and positive spatial correlations were used to visually represent (i.e., map) mortality rates of non-alcoholic liver disease and cyanobacterial bloom coverage by county in the United States.

Shriber et al. [56] developed a countywide vulnerability index based on sociodemographic and health status variables to determine susceptibility to the fungal infection coccidioidomycosis. The methodology used principal component analysis (PCA) with weighted and unweighted variables, which included exposure variables and those of human health sensitivity and adaptive capacity, to assess the health risk of acquiring coccidioidomycosis due to changes in climate, specifically temperature, precipitation, and drought index. Only Shriber et al. [56] and Zhang et al. [62] incorporated health measures into their analyses. The remaining studies used environmental variables and vector locations to make associations between climate and vector-borne disease transmission.

### 3.4. Wildfires

Only one of the papers reviewed (3% of the selected studies) examined wildfires and bushfires and their connection to adverse health outcomes [38]. In their analysis, Gaither et al. [38] examined the exposure to smoke plume conditions in the Southeastern United States and calculated a social vulnerability index. These conditions were compiled from satellite data accessed via the NOAA Hazard Mapping System (HMS). This data was then aggregated to define areas in which smoke plumes occurred and where they intersected census block groups in the study area. A social vulnerability index was calculated by totaling each individual vulnerability indicator proportion (by population at the census block group) and dividing by the total number of variables assessed. The authors found that cardiopulmonary illnesses were positively associated with higher vulnerability, although they were unable to obtain specific health outcome data. Variables assessed included poverty status, those aged 65 years and older, and those 15 years and younger—populations that are more susceptible to the ill effects of air pollutants.

## 4. Discussion

This review examined thirty-seven papers that studied at-risk populations and described spatial vulnerability in terms of sociodemographic characteristics, health status, and environmental factors and how they affect human health outcomes associated with climate change. All papers had a similar approach: they identified a climate exposure (e.g., high temperatures) and then determined which populations had or may be susceptible to negative health outcomes due to that exposure. Data were obtained to represent the environmental variables (e.g., daily temperature data) and variables that measured population sensitivity and adaptive capacity (e.g., age and poverty status or distance to hospitals) for a specific geographic location. These data were then mapped to visually represent current or future at-risk populations. While there has been an expansion in the use of vulnerability maps to assess health outcomes to climate change, as they present complex information in a simplified format that many individuals can understand [68], there are still limitations, specifically around the types of vulnerability maps and the data available to produce these maps. Methodologies used tend to be more academic and less accessible to public health professionals working on identifying vulnerability and building adaptation to climate change, though as technology becomes more accessible this will change [27]. Additionally, frameworks for assessing vulnerability have been established for some time [6,7], though there are still improvements which are required to translate vulnerability assessment frameworks into tools that can be used to develop vulnerability maps. A key limitation to those improvements has been a lack of data availability, particularly with health outcome data [15,69].

Eriksen and Kelly [14] emphasize that when selecting variables, or indicators, for mapping vulnerability, it is important to capture more than just a snapshot of one moment in time. In a number of studies reviewed, simple overlays of vulnerable populations and potential exposures did not include a temporal component of vulnerability. Aspects of social vulnerability are multidimensional and while researchers try to linearly extrapolate data, population over time and geographic area change in non-linear ways. It is worthwhile, though time-consuming, to capture the dynamic aspects of the variation of vulnerability over time and space to reduce uncertainty in projecting vulnerability [27].

In many of the studies, proxies for health (e.g., poverty status, age, and disability) were obtained from census data and used in vulnerability indices and maps to identify populations that may have increased risks to health associated with climate hazards. In some cases, data were requested from local public health agencies or hospitals with personal identifiable information removed. The use of proxy data to identify health vulnerabilities were employed in a number of studies. Some studies included prevalence of health conditions in a vulnerability index [15,20,21,36,48,51], while others looked specifically at mortality as an outcome [19,39,40,41,43,44,47,52,57,62], and a few examined specific health morbidity outcomes via maps [42,48]. While satellite data and hazard exposure data may be available at finer scales, health data is most often the determinant of spatial scale for the analysis. Proxies, such as the ones used in the studies examined, may be the best representation of health-related data available to researchers since they are widely available. Although it may reduce the sensitivity of the analysis, they provide important health-related information on population level vulnerability to climate hazards. Additionally, engaging with stakeholders such as community-based organizations or other non-for-profit entities that work closely with communities in the geographic area to be mapped can potentially provide finer scale data related to health outcomes. Ideally access to individual health data would greatly improve our understanding of the associations between specific health outcomes related to climate change. Health data also differs outside the U.S. with many European and Asian countries having universal healthcare systems. Having access to health data on larger segments on the population may remove the need for proxies and help identify sensitivities as well and may be an advantage in these locations outside the U.S.

Researchers should exemplify how variables measuring the sensitivity and adaptive capacity of different populations help to determine the potential for climate-related hazards effect on human health. Sensitivity and adaptive capacity also represent current, not future, conditions and add another level of uncertainty when determining spatial scale for future projections [25,69]. By addressing the methodology, data gaps, sensitivity, and potential for adaptive capacity vulnerability maps can become comparable across scales. Generalizability varies with scales, and though studies use standardized vulnerability indices, variables chosen to examine vulnerability did not always appear across all studies examined in this review. Future directions for research in vulnerability mapping will benefit from standardized definitions of exposure, sensitivity, and adaptive capacity to climate hazards. Whether it is a heat wave temperature threshold, hospitalization data, or air conditioner use, establishing the spatial extent at which sociodemographic, health, and environmental variables can be defined is key. Assessing these details, can contribute to the vulnerability assessment narrative and potentially direct adaptation resources toward their most efficient and effective use. The following discussion identifies limitations and recommendations that can improve our efforts to produce vulnerability maps that work toward this goal.

Scale is arguably one of the most important elements when preparing vulnerability maps. The scale of a map can represent vulnerability from the global level down to the neighborhood level, based on data availability, with finer resolutions offering greater sensitivity across populations. As satellite images and processing technology improve, mapping the geographic and topographic landscape is improving. Unfortunately, this progress may be hampered by a lack of corresponding socioeconomic and health data, which are used to estimate the associations between the changing physical environment and human health vulnerability to climate hazards. To address the issue of scale, researchers need to first examine the data we use to produce maps. Vulnerability happens at multiple scales and requires the selection of representative vulnerability indicators across varying extents and resolutions. It is desirable to express vulnerability at the individual and local extents and at fine-scale resolution. This requires researchers to look at the data and assess a number of questions, for instance, are census blocks the preferred resolution at which to do vulnerability analysis in the United States? How do measures of climate hazard affect the scale at which local vulnerability is reported? Does the impacted community have viable data at this level? These questions should be addressed at the outset to establish the best approaches for developing vulnerability maps. Data that measure sensitivity and adaptive capacity, particularly health data, usually determine scale and obtaining this at the finest resolution possible will better inform individual and local vulnerability.

While county-level health statistics are sometimes available, these are also limited and may not accurately reflect health outcomes associated with climate hazards. In the United States, incidence of disease is often aggregated to comply with health privacy laws. Although, secondary data used in epidemiological research is routinely collected by hospitals and local health departments, confidentiality and restrictions on the use of individual health data limit the scalability of certain analyses to protect privacy [25,70]. To address this issue connected to scale, is the array of vulnerability indicators that may be available. By engaging in an examination of all possible indicators, researchers can attempt to fill gaps by using proxies to represent the sensitivity and adaptive capacity needed to measure health outcomes. Obtaining access to health records or hospitalization data help to directly tie-in how climate change affects health outcomes, but using social determinants of health as proxies, which are supported by epidemiological evidence, can provide information on the sensitivity of population.

De Sherbinin [25] stresses a point made by Kok et al. [71] that a significant gap exists between vulnerability assessments conducted at the local level, and those that are done at a global or national scale which are based on aggregated data and “crude” underlying assumptions. Differences in hazard definition, representation of the variables that define vulnerability, and methodological differences have been observed. Limited data availability for human health outcomes is an obstacle that prevents researchers from establishing clear exposure-response relationships for climate-related health hazards. Anecdotally, we know that instances of high precipitation may increase risks of water-borne disease or that an increase in the habitat of vector-borne disease carriers may increase the likelihood of certain vector-borne diseases associated with climate hazards (e.g., malaria). Improving data collection, especially enhanced surveillance of human health after exposure to climate hazards, may improve researchers’ ability to estimate statistical associations. In the U.S. and globally, applying common hazard definition will also help researchers and government officials identify threats to vulnerable populations. For example, while a temperature of 90F may be exceed averages in San Francisco, California, it may be commonplace in New Delhi, India. Methods for standardizing definitions help to compare events across geographies and enhance our understanding of population sensitivities. In addition, including a temporal indicator for health outcomes will enhance the dynamic nature of vulnerability maps, allowing for better current and future projections of population vulnerability and reducing uncertainty of underlying assumptions.

Maps are also an effective tool in the communication or visualization of data. A goal for vulnerability mapping is to clearly define geographic and sociodemographic vulnerability to certain exposures. Once these have been defined, to enhance the communication of the data engagement with the community is key to achieve buy-in and potentially assess data collection from within the affected community. Additionally, care should be taken to avoid overloading mapped data in such a way as to render it useless to non-scientific audiences or end users. Simplicity, whether it is in a single map or across multiple maps, will help to convey the best information available to support decision-making. Maps convey information beyond a single language and can help to communicate information among populations across multiple geographies. Health also ties individuals to climate change. Involving stakeholders and individuals whose health is, or may be, affected by climate change give them more reason to become involved in helping to reduce their vulnerability. Feedback from stakeholders or from the end users of the maps will help to better prepare and design these ‘pictures’ of vulnerability.

## 5. Conclusions

If the frequency and intensity of extreme events associated with climate change continue to increase, describing and quantifying vulnerable populations, specifically those who experience an undue burden to health, will be especially important for identifying and adopting future adaptation strategies. This review suggests that research in the US is moving in the right direction, but much more needs to be done. Globally there has been advancement in mapping vulnerability to other climate-related hazards, such as vector-borne diseases. Exploring these, and other methodologies and data sets used to study the health impacts of climate related extreme events highlight ways in which we can overcome certain challenges, including issues of scale, analysis, and data availability. Future research directions should include studies of vulnerability mapping, not just in the United States, but around the globe to better understand methodologies and data used in various geographic areas. The insight gleaned from vulnerability mapping used, for example, in Sub-Saharan Africa, Southeast Asia, or Western Europe may help to elucidate innovative vulnerability mapping techniques which can be applied to different geographies and improve identification of population sensitivities to climate-related hazards. Improving our surveillance of climate-related illnesses will also increase our ability to use more specific and local health data to better inform vulnerability maps. Vulnerability maps are also communication tools that can engage with stakeholders and individuals to understand their vulnerability and ultimately reduce negative health outcomes associated with climate-related hazards. The recommendations provided in this review aim to help the vulnerability map-making process by linking health outcomes to climate-related hazards to improve researchers understanding of how scale and vulnerability indicators can help in establishing methodologies that are appropriate and robust enough to satisfy the analytical requirements and produce maps that can help us identify vulnerable populations and build resilience within these communities.

## Figures and Tables

**Table 1:** Characteristics of studies examining vulnerability mapping of human health to climate change.

Study	Location	Exposure	Measures of Exposure, Sensitivity, and Adaptive Capacity	Measure/Assessment of Health Vulnerability
Aubrecht and Özceylan (2013) [31]	Washington Metropolitan Area (National Capital Region)	High ambient temperature	Exposure (environmental) variables: GHCN (Global Historical Climatology Network); U.S. Geological Survey (USGS) National Land Cover Database 2006 Sensitivity and/or Adaptive Capacity variables: U.S. Census and the American Community Survey (ACS) and U.S. Geological Survey (USGS) National Land Cover Database	Heat-stress vulnerability index (HVI)
Boumans et al. (2014) [32]	Travis County, Texas	High ambient temperature	Exposure (environmental) variables: U.S. Geological Survey (USGS) National Land Cover Database 2001; Bjerknes Centre for Climate Research-Bergen Climate Model Version 2 Sensitivity and/or Adaptive Capacity variables: U.S. Census	Heat-stress vulnerability index (HVI)
Bradford et al. (2015) [33]	Pittsburg, Pennsylvania	High ambient temperature	Exposure (environmental) variables: U.S. Geological Survey (USGS) National Land Cover Database Sensitivity and/or Adaptive Capacity variables: U.S. American Community Survey (2008–2012); Behavioral Risk Factor Surveillance System; Allegheny County Tax Property Office	Heat vulnerability index (HVI)
Burton and Cutter (2008) [34]	The Sacramento - San Joaquin Delta	Flood hazard	Exposure (environmental) variables: FEMA’s Hazus MH 2 flood model Sensitivity and/or Adaptive Capacity variables: U.S. Census and the American Community Survey (ACS)	Social vulnerability index for floods (levee failure)
Chow et al. (2012) [35]	Phoenix, Arizona	High ambient temperature	Exposure (environmental) variables: Temperature data from 37 local meteorological stations. Landsat ETM+ data Sociodemographic variables: U.S. Census	Heat stress vulnerability index
Chuang and Gober (2015) [36]	Phoenix, Arizona	High ambient temperature	Exposure (environmental) variables: NASA Land Processes Distributed Active Archive Center Sensitivity and/or Adaptive Capacity variables: Arizona Department of Health Services’ hospital discharge databases for 2004 and 2005; U.S. Census; Maricopa County Assessor’s Office	Heat vulnerability index for hospitalizations
Cleckner and Allen (2014) [37]	Chesapeake, Virginia	Vector-borne disease	Exposure (environmental) variables: NOAA’s Coastal Change Analysis Program; Mosquito abundance values (Sutherest et al. 2004, Cleckner et al. 2011) Sensitivity and/or Adaptive Capacity variables: U.S. Census; City of Chesapeake IT Department	Spatial Vulnerability index calculated for mosquito exposure
Gaither et al. (2015) [38]	Southern, US (13 States)	Wildfires	Exposure (environmental) variables: NOAA’s Hazard Mapping System (HMS); Southern Forest Futures Assessment and Climate Change Adaptation and Mitigation Management Options Sensitivity and/or Adaptive Capacity variables: U.S. Census	Social vulnerability index (SOVU)
Harlan et al. (2013) [39]	Maricopa County, Arizona	High ambient temperature	Exposure (environmental) variables: Landsat ETM+ data—NASA Sensitivity and/or Adaptive Capacity variables: U.S. Census; Tax Assessor’s 2010 parcel registry; Maricopa County Department of Public Health	Heat vulnerability index (HVI)
Hattis et al. (2012) [40]	Massachusetts	High ambient temperature	Exposure (environmental) variables: NOAA’s National Climate Data Center Sensitivity and/or Adaptive Capacity variables: U.S. Census; Massachusetts Department of Public Health’s Registry of Vital Records and Statistics	Heat mortality
Heaton et al. (2014) [41]	Houston, Texas	High ambient temperature	Exposure (environmental) variables: Noah LSM—High Resolution Land Data Assimilation System Sensitivity and/or Adaptive Capacity variables: U.S. Census; Texas Department of State Health Services; Harris County Appraisal District	Heat mortality
Holt et al. (2009) [42]	California	Vector-borne disease	Exposure (environmental) variables: California Department of Health and the United States Department of Agriculture/Wildlife Services; Worldclim bioclimatic variables Sensitivity and/or Adaptive Capacity variables: California Department of Public Health	Vector-borne disease: Plague
Hondula et al. (2012) [43]	Philadelphia County, Pennsylvania	High ambient temperature	Exposure (environmental) variables: Landsat ETM+ data—NASA; Zoning and Land Use data (PASDA) Sensitivity and/or Adaptive Capacity variables: U.S. Census; NHGIS (National Historical Geographic Information System)	Heat-related mortality.
Hondula et al. (2015) [44]	Atlanta, Boston, Minneapolis-St. Paul, Philadelphia, Phoenix, Seattle, St. Louis	High ambient temperature	Exposure (environmental) variables: NOAA National Climatic Data Center; U.S. Geological Survey (USGS) National Land Cover Database Sensitivity and/or Adaptive Capacity variables: U.S. Census; NHGIS (National Historical Geographic Information System)	Heat mortality
Huang et al. (2011) [45]	Gwynns Falls Watershed, Baltimore Counties, Maryland	High ambient temperature	Exposure (environmental) variables: Landsat ETM+ data—NASA Sensitivity and/or Adaptive Capacity variables: U.S. Census; Applied Geographic Solution’s “CrimeRisk” database for total crime index	Socially vulnerable ‘hotspots’
Johnson et al. (2009) [19]	Philadelphia, Pennsylvania	High ambient temperature	Exposure (environmental) variables: Landsat TM 5 data—NASA; U.S. Geological Survey (USGS) National Land Cover Database Sensitivity and/or Adaptive Capacity variables: U.S. Census; Pennsylvania Department of Health	Heat-related mortality
Johnson et al. (2012) [18]	Chicago, Illinois	High ambient temperature	Exposure (environmental) variables: Landsat TM 5 data—NASA; U.S. Geological Survey (USGS) National Land Cover Database Sensitivity and/or Adaptive Capacity variables: U.S. Census; Illinois State Vital Records Department	Extreme Heat vulnerability index (EHVI)
Johnson et al. (2013) [46]	Chicago, Illinois; Dayton, Ohio; Indianapolis, Indiana	High ambient temperature	Exposure (environmental) variables: Landsat TM 5 data—NASA; U.S. Geological Survey (USGS) National Land Cover Database Sensitivity and/or Adaptive Capacity variables: U.S. Census; NASA’s Research Opportunities in Space and Earth Sciences (ROSES)—heat mortality	Extreme Heat vulnerability index
Klein-Rosenthal et al. (2014) [47]	New York, New York	High ambient temperature	Exposure (environmental) variables: Landsat 7 ETM—NASA; U.S. Forest Service Sensitivity and/or Adaptive Capacity variables: U.S. Census; NYC DOHMH Office of Vital Statistics; NYC Dept. of City Planning; NYC Dept. of Housing Preservation & Development; NYC Dept. of Finance	Heat mortality
Kovach et al. (2015) [48]	North Carolina	High ambient temperature	Exposure (environmental) variables: USDA-NASS Cropland Data Layer; U.S. Geological Survey (USGS) National Land Cover Database Sensitivity and/or Adaptive Capacity variables: U.S. Census; North Carolina Disease Event Tracking and Epidemiologic Tool	Heat-related morbidity
Liu and Weng (2012) [49]	Los Angeles County, California	Vector-borne disease	Exposure (environmental) variables: NASA—Terra ASTER and MODIS; U.S. Geological Survey Digital Elevation Model Sensitivity and/or Adaptive Capacity variables: California Department of Public Health; UC Davis Center for Vector-borne Diseases; California Department of Food and Agriculture; Mosquito and Vector Control Association of California.	West Nile Virus (WNV) ‘risk areas’
Liu et al. (2015) [50]	Gloucester, Isle of Wight, Matthews, and York Counties, Virginia	Flood hazard	Exposure (environmental) variables: U.S. Army Corps of Engineers SLOSH model; U.S. Geological Survey National Elevation Dataset (NED) Sensitivity and/or Adaptive Capacity variables: Household vulnerability survey (Random sample)	Household vulnerability index
Maantay et al. (2010) [51]	New York, New York	Flood hazard	Exposure (environmental) variables: FEMA Q3 100-year floodplain Sensitivity and/or Adaptive Capacity variables: U.S. Census; NYS SPARCS FEMA Q3 100-year floodplain—flood hazard	Hazard vulnerability index (HazVI) Combined with enhanced spatial methodology
Madrigano et al. (2015) [52]	New York, New York	High ambient temperature	Exposure (environmental) variables: NOAA—National Climatic Data Center; Landsat TB data—NASA Sensitivity and/or Adaptive Capacity variables: U.S. Census; NYC DOHMH Office of Vital Statistics; NYC Department of City Planning	Heat mortality index
Maier et al. (2014) [20]	Georgia	High ambient temperature	Exposure (environmental) variables: NOAA—National Climatic Data Center; Landsat TB data—NASA Sensitivity and/or Adaptive Capacity variables: U.S. Census; CDC Behavioral Risk Factor Surveillance System; University of Georgia Natural Resources Spatial Analysis Lab; National Center for Health Statistics	Heat vulnerability index (HVI)
Manangan et al. (2014) [15]	Georgia	High ambient temperature	Exposure (environmental) variables: U.S. Geological Survey (USGS) National Land Cover Database; CDC National Environmental Health Tracking program Sensitivity and/or Adaptive Capacity variables: U.S. Census; Centers for Medicare and Medicaid Services; Homeland Security Infrastructure Program	Heat vulnerability index
Martinich et al. (2012) [53]	Coastline of continental United States	Flood hazard	Exposure (environmental) variables: Sea level rise, National Coastal Property Model—EPA Sensitivity and/or Adaptive Capacity variables: U.S. Census and the American Community Survey (ACS)	Social vulnerability index (SoVI)
Méndez-Lázaro et al. (2018) [54]	San Juan, Puerto Rico	High ambient temperature	Exposure (environmental) variables: USGS Landsat 8 Operational Land Imager—Thermal Infrared Sensor Puerto Rico Terrestrial Gap Analysis Project—USDA Sensitivity and/or Adaptive Capacity variables: U.S. Census and the American Community Survey (ACS)	Heat vulnerability index (HVI)
Prudent et al. (2016) [55]	Travis County, Texas	High ambient temperature & Flood Hazard	Exposure (environmental) variables: Landsat 5 & 7 ETM—NASA; Impervious surfaces—USGS; FEMA Q3 100-year floodplain; Low-water crossing—Austin, TX Watershed Department Sensitivity and/or Adaptive Capacity variables: U.S. Census; Texas Department of State Health Services	Social-built environment index
Reid et al. (2009) [21]	United States	High ambient temperature	Exposure (environmental) variables: U.S. Geological Survey (USGS) National Land Cover Database Sensitivity and/or Adaptive Capacity variables: U.S. Census; CDC Behavioral Risk Factor Surveillance System; American Housing Survey	Heat vulnerability index (HVI)
Shriber et al. (2017) [56]	Arizona & California	Infectious micro-organism	Exposure (environmental) variables: Multi-Resolution Land Characteristics Consortium (2011); Skin Test (Edwards & Palmer 1957) Sensitivity and/or Adaptive Capacity variables: U.S. Census; CDC BRFSS; National Cancer Institute; American Hospital Association; HRSA Area Health Resource File	Coccidioiomycosis Index
Uejio et al. (2011) [57]	Philadelphia, Pennsylvania and Phoenix, Arizona	High ambient temperature	Exposure (environmental) variables: NASA’s ASTER (Advanced Space-borne Thermal Emission and Reflection Radiometer) Sensitivity and/or Adaptive Capacity variables: U.S. Census	Heat mortality (Philadelphia) Heat Distress Calls (Phoenix)
Wang and Yarnal (2012) [58]	Sarasota, Florida	Flood hazard	Exposure (environmental) variables: U.S. Army Corps of Engineers SLOSH model; FEMA flood insurance rate maps Sensitivity and/or Adaptive Capacity variables: U.S. Census and the American Community Survey (ACS)	Social vulnerability indicators (SoVI)
Weber et al. (2015) [59]	Philadelphia, Pennsylvania	High ambient temperature	Exposure (environmental) variables: NOAA National Climatic Data Center; NASA Moderate Resolution Imaging Spectroradiometer (MODIS) Sensitivity and/or Adaptive Capacity variables: U.S. Census	Social vulnerability index
Wilson and Chakraborty (2018) [60]	Chicago, Illinois	High ambient temperature	Exposure (environmental) variables: NASA Moderate Resolution Imaging Spectroradiometer (MODIS) Sensitivity and/or Adaptive Capacity variables: U.S. Census; Neighborhood Change Database—Geolytics;	Heat vulnerability index (HVI)
Wisconsin Department of Health Services (2014) [61]	Milwaukee, Wisconsin	High ambient temperature	Exposure (environmental) variables: Oregon State/ U.S. Geological Survey (USGS)—Parameter-elevation Regressions on Independent Slopes Model (PRISM) and National Land Cover Database; EPA Air Quality Index Sensitivity and/or Adaptive Capacity variables: U.S. Census; Wisconsin Department of Health Services; Milwaukee County Behavioral Health Division; Wisconsin Division Long Term Care	Heat vulnerability index (HVI)
Zhang et al. (2015) [62]	United States	Infectious micro-organism	Exposure (environmental) variables: NASA The Medium Resolution Imaging Spectrometer (MERIS) Sensitivity and/or Adaptive Capacity variables: U.S. Census; CDC’s Wide-ranging Online Data for Epidemiologic Research	Liver disease mortality

Abbreviations: FEMA—U.S. Federal Emergency Management Agency; NASA—U.S. National Aeronautics and Space Administration; NOAA—U.S. National Oceanic and Atmospheric Administration; LSM—Land Surface Model; ASTER—Advanced Spaceborne Thermal Emission and Reflection Radiometer; MODIS—Moderate Resolution Imaging Spectroradiometer; NYS SPARCS—New York State—Statewide Planning and Research Cooperative System; EPA—U.S. Environmental Protection Agency; CDC—U.S. Centers for Disease Control & Protection.
